# Antimicrobial and Anticancer Activities of *Lactiplantibacillus plantarum* Probio87 Isolated from Human Breast Milk

**DOI:** 10.3390/nu17152554

**Published:** 2025-08-05

**Authors:** Pei Xu, Mageswaran Uma Mageswary, Azka Ainun Nisaa, Xiang Li, Yi-Jer Tan, Chern-Ein Oon, Cheng-Siang Tan, Wen Luo, Min-Tze Liong

**Affiliations:** 1 College of Culinary and Food Science Engineering, Sichuan Tourism University, Chengdu 610100, China; xupei386@gmail.com (P.X.); shiclx@163.com (X.L.); 2 School of Industrial Technology, Universiti Sains Malaysia, Gelugor 11800, Malaysia; umamageswary@student.usm.my (M.U.M.); azka.nisaa@gmail.com (A.A.N.); 3 Institute for Research in Molecular Medicine, Universiti Sains Malaysia, Gelugor 11800, Malaysia; richtan54@gmail.com (Y.-J.T.); chern.oon@usm.my (C.-E.O.); 4 Faculty of Medicine and Health Sciences (FMHS), Universiti Malaysia Sarawak (UNIMAS), Kota Samarahan 94300, Malaysia; cstan@unimas.my

**Keywords:** *Lactiplantibacillus plantarum*, probiotic properties, antimicrobial activity, anticancer, cervical cancer cells, *in vitro*

## Abstract

**Background/Objectives:** This study evaluated the *in vitro* probiotic potential of *Lactiplantibacillus plantarum* Probio87 (Probio87), focusing on its physiological robustness, safety, antimicrobial properties, and anticancer activity, with relevance to vaginal and cervical health. **Methods:** Tests included acid and bile salt tolerance, mucin adhesion, and carbohydrate utilization. Prebiotic preferences were assessed using FOS, GOS, and inulin. Antibiotic susceptibility was evaluated per EFSA standards. Antimicrobial activity of the cell-free supernatant (CFS) was tested against *Staphylococcus aureus*, *Escherichia coli*, and *Candida* species. Effects on *Lactobacillus iners* and *L. crispatus* were analyzed. Anticancer properties were assessed in HeLa, CaSki (HPV-positive), and C-33A (HPV-negative) cervical cancer cell lines through proliferation, apoptosis, angiogenesis, and cell cycle assays. **Results:** Probio87 showed strong acid and bile tolerance, efficient mucin adhesion, and broad carbohydrate utilization, favoring short-chain prebiotics like FOS and GOS over inulin. It met EFSA antibiotic safety standards. The CFS exhibited potent antimicrobial activity, including complete inhibition of *Candida albicans*. Probio87 selectively inhibited *L. iners* without affecting *L. crispatus*, indicating positive modulation of vaginal microbiota. In cervical cancer cells, the CFS significantly reduced proliferation and angiogenesis markers (*p* < 0.05), and induced apoptosis and cell cycle arrest in HPV-positive cells, with minimal effects on HPV-negative C-33A cells. **Conclusions:** Probio87 demonstrates strong probiotic potential, with safe, selective antimicrobial and anticancer effects. Its ability to modulate key microbial and cancer-related pathways supports its application in functional foods or therapeutic strategies for vaginal and cervical health.

## 1. Introduction

Probiotics are defined by the Food and Agriculture Organization (FAO) and the World Health Organization (WHO) as live microorganisms that confer health benefits when consumed in adequate amounts [1]. FAO and WHO guidelines outline key probiotic criteria, including resistance to gastric acidity and bile salts, adherence to mucosal surfaces, and the absence of antibiotic resistance [2]. Understanding the ability of probiotics to utilize prebiotics is also crucial, as it influences gut microbiota composition and overall gastrointestinal health [3]. In addition, the cell-free supernatant (CFS), which contains metabolites produced by probiotic strains, has been reported to exhibit antimicrobial properties due to the presence of bioactive compounds [4].

*Lactobacillus* species are widely reported to possess probiotic properties and a long history of safe use in human history [5]. *Lactiplantibacillus plantarum*, formerly known as *Lactobacillus plantarum*, is a Gram-positive lactic acid bacteria (LAB) commonly found in fermented foods and the gastrointestinal tract (GIT). It is widely used in the food industry as a probiotic due to its diverse health benefits, including antioxidant, anticancer, anti-inflammatory, antiproliferative, anti-obesity, and anti-diabetic properties [6]. Additionally, studies suggest its potential to enhance cognitive function in individuals with major depression [7] and alleviate stress and anxiety in adults [8].

Human papillomavirus (HPV) is a small, non-enveloped, double-stranded DNA virus. Low-risk HPV types are associated with benign warts [9,10], whereas high-risk HPV types, particularly HPV 16 and 18, are strongly linked to cervical cancer [11]. Persistent infection with HPV 16 or 18 is responsible for 70% of cervical cancers and 50% of CIN 3 lesions [12]. Probiotics have demonstrated antiviral properties through various mechanisms, including immunomodulation [13]. They have shown activity against multiple viruses, such as human rhinoviruses, enteroviruses, influenza viruses, respiratory syncytial viruses, adenoviruses, parainfluenza viruses, and coronaviruses. As natural, non-drug alternatives, probiotics modify innate immunity and regulate pathogen-induced inflammation via toll-like receptor signaling pathways [14,15], indicating their potential benefit for HPV clearance.

This study evaluated the probiotic properties of *Lactiplantibacillus plantarum* Probio87, including its acid and bile tolerance, mucin adherence, antibiotic susceptibility, antimicrobial activity, symbiotic properties, and prebiotic utilization, to evaluate its suitability as a potential probiotic for human consumption. Additionally, its inhibitory effect on cervical cancer cell lines, including both HPV-positive and HPV-negative cells, was examined. These findings suggest that Probio87 may be a promising probiotic candidate for inhibiting HPV infection.

## 2. Materials and Methods

### 2.1. Probiotic Properties

#### 2.1.1. Strains and Culture Conditions

*Lactiplantibacillus plantarum* Probio87 (Probio87) was obtained courtesy of Probionic Corp., Republic of Korea. All other microorganisms were obtained from the American Type Culture Collection (ATCC): *Lactobacillus crispatus* (ATCC 33820), *Lactobacillus iners* (ATCC 55195), *Escherichia coli* (ATCC 11775), *Staphylococcus aureus* (ATCC 12600), *Candida albicans* (ATCC 18804), *Candida glabrata* (ATCC 2001), *Candida krusei* (ATCC 6258), *Candida parapsilosis* (ATCC 22019), and *Candida tropicalis* (ATCC 750). Bacterial strains were identified by 16S rRNA sequencing, and yeast species were confirmed by 18S rDNA sequencing prior to use.

All strains were sub-cultured three times for 24 h at 37 °C before the experiments to ensure activation. *Lactobacillus iners* was cultivated in a modified Brain Heart Infusion (BHI) broth (BD, Franklin Lakes, NJ, USA) supplemented with 1% (*w*/*v*) yeast extract (BD), 0.1% (*w*/*v*) maltose (Sigma-Aldrich, St. Louis, MO, USA), 0.1% (*w*/*v*) glucose (Sigma-Aldrich), and 10% (*v*/*v*) horse serum (Thermo Fisher Scientific, Waltham, MA, USA) [16]. Other *Lactobacillus* strains including Probio87 were cultured in sterile de Mann, Rogosa, Sharpe (MRS) broth (HiMedia, Mumbai, India), while *Candida* species were cultured in yeast extract peptone dextrose (YEPD) medium, which contained 1% (*w*/*v*) yeast extract (Himedia, Mumbai, India), 2% (*w*/*v*) peptone, and 2% (*w*/*v*) dextrose (Bendosen, Selangor Darul Ehsan, Malaysia). *Staphylococcus aureus* and *Escherichia coli* were activated and cultured in Tryptic Soy Broth (TSB; Oxoid, Basingstoke, UK).

#### 2.1.2. Cell-Free Supernatant (CFS) Preparation

All stock cultures were preserved in 20% glycerol at −20 °C. Prior to use, they were activated by incubating at 37 °C for 24 h in the respective culture media, with 10% (*v*/*v*) inoculum, for three successive cycles. The activated cultures were diluted with culture medium to an optical density of 1.0 at 600 nm (10^8^ CFU/mL), then centrifuged at 12,000× *g* for 5 min at 4 °C. The supernatant was collected, neutralized to pH 7.0, filter-sterilized, and stored at −80 °C for future analysis.

#### 2.1.3. Carbohydrate Utilization

The API 50 CHL kit (BioMérieux, Marcy l’Etoile, France) was used following the manufacturer’s guidelines. Single colonies from the Probio87-cultivated MRS agar plate were combined with 1 mL of sterile water to create a thick suspension, which was transferred to 5 mL of sterile water to achieve a 2 McFarland standard, noting the number of drops added. The 10 mL 50 CHL Medium ampule (BioMérieux) was opened, and twice the number of drops of the bacterial suspension were added to create a 2 McFarland mixture. The mixture was then transferred into each strip tube, avoiding bubbles, and sealed with mineral oil (Acros Organics, Fair Lawn, NJ, USA). The strips were placed in an inoculation tray with 10 mL of distilled water, covered with the provided plastic lid, and incubated horizontally at 37 °C. After 24 and 48 h, the color of each tube was checked. A yellow or black color (tube 25) indicated a positive result, while purple indicated a negative result. If a positive result turned negative at 48 h, only the initial reading was considered.

#### 2.1.4. Acid Tolerance and Bile Tolerance

The acid tolerance method was adapted from [17] with modifications, the pH of MRS broth was adjusted to 1.5, 2.5, 3.5, 4.5, 5.5, and 7 using 1M hydrochloric acid (Merck, Rahway, NJ, USA), while the bile tolerance test was conducted following the method [18] with slight modifications. MRS broths with varying bile concentrations (0%, 0.1%, 0.2%, 0.3%, 0.4% and 0.5% *w*/*v*) were prepared. An overnight activated Probio87 suspension with an optical density of 1 ± 0.05 at 600 nm was inoculated at 2% (*v*/*v*) into the pH-adjusted broths and 1% (*v*/*v*) into each bile salt broth, then incubated at 37 °C for 4 h. After incubation, the broths were mixed and serially diluted 10-fold in MRS broth. Subsequently, 100 μL of each dilution was mixed with 15 mL of MRS agar (HiMedia, Mumbai, India) and poured into sterile Petri dishes. These plates were anaerobically incubated for 48 h under normal conditions, followed by colony counting. The viability of the strains in different broths was assessed by comparing the colony counts (CFU/mL) in the treated broths (*N_t_*) to those in the control broth (*N*_0_) using Equation (1) [8,19].

(1) $$Survival\;rate\left(\%\right)=\frac{\mathrm{lg}\left(N_{t}\right)}{\mathrm{lg}\left(N_{0}\right)}\times 100\%$$

#### 2.1.5. Antibiotic Susceptibility

The assay followed a modified broth microdilution method [20], using LAB susceptibility test medium (LSM) to support the growth of Probio87. LSM is a mixture of 90% (*v*/*v*) Iso-Sensitest broth (OXOID, Hampshire, UK) and 10% (*v*/*v*) MRS broth, adjusted to pH 6.7. The antibiotic susceptibility was assessed following the Clinical and Laboratory Standards Institute (CLSI) M45 broth microdilution method [21]. Briefly, the isolated colonies were suspended in sterile saline solution (Bendosen, Selangor Darul Ehsan, Malaysia) and adjusted to a turbidity of a 0.5 McFarland standard. The suspension was then diluted 1:150 in LSM broth to prepare the inoculum. Next, 100 μL of diluted antibiotic solution was added to each well of the first column in a 96-well plate and two-fold serial dilutions up to the 11th column. The 12th column, containing only culture medium, served as the growth control. Finally, 50 μL of the prepared inoculum was added to each well. The plate was scanned at 625 nm to obtain the initial OD reading and then incubated at 37 °C for 18 h before taking the final OD reading to get the minimum inhibitory concentrations (MICs).

#### 2.1.6. Prebiotic Utilization

A medium similar to MRS was prepared (Table 1), substituting glucose with fructooligosaccharide (FOS), galactooligosaccharide (GOS), and inulin, following a previously established protocol [8]. The activated Probio87 strain was washed twice with PBS and suspended in the prepared solutions. The suspension was adjusted to an OD of 0.3 ± 0.02 at 600 nm, and 100 μL was mixed with an equal volume of the corresponding medium in a 96-well plate. The plate was incubated under standard conditions and scanned at 600 nm every 2 h for 24 h.

#### 2.1.7. Adherence to Mucin

The method outlined by Tham et al. [22] and Carasi et al. [23] was used with minor modifications. Each well of a 96-well plate was coated with 100 μL of 10 mg/mL solution of porcine gastric mucin (type III, partially purified) (Sigma-Aldrich, St. Louis, MO, USA). Excess mucin was aspirated, and the wells were washed twice with 200 μL of sterile PBS. The activated strain suspension was adjusted to an OD_600_ of 0.5 ± 0.02. Then, 100 μL of this suspension was added to the 96-well plate and incubated at 37 °C for 3 h. The wells were washed five times with 200 μL of PBS before being treated with 200 μL of 0.05% Triton-X-100 solution (Sigma-Aldrich) at 25 °C for 10 min [24]. The contents were mixed, and 100 μL was serially diluted 10-fold with PBS. Then, 100 μL of these dilutions were mixed with 15 mL of MRS agar (HiMedia) and plated. Colonies were counted after anaerobic incubation at 37 °C for 48 h. The adhesion rate was determined using Equation (2) from Pabari et al. [25], where *N_t_* is the number of colonies (CFU/mL) after adhesion, and *N*_0_ is the colony count before adhesion.

(2) $$\mathrm{Relative}\mathrm{adhesion}\;(\%)=\frac{\mathrm{lg}\left(N_{t}\right)}{\mathrm{lg}\left(N_{0}\right)}\times 100$$

### 2.2. Antimicrobial Properties

#### 2.2.1. Inhibition Against Common Pathogens

The antimicrobial effectiveness of Probio87 against *S*. *aureus* and *E*. *coli* was assessed using the method from [26]. Briefly, pathogens were cultured overnight in TSB (OXOID) at 37 °C and then adjusted to OD_600_ 0.3 ± 0.02 with TSB. A 100 µL suspension of each pathogen was mixed with an equal volume of CFS from Probio87 in a 96-well microplate. The mixture was incubated at 37 °C for 24 h, with OD_600_ readings taken every 4 h. MRS broth served as the negative control, and Streptomycin (10 µg/mL) was used as the positive control for *S*. *aureus* and Amoxicillin (1.5 μg/mL) for *E*. *coli* [8].

#### 2.2.2. Inhibition Against Pathogenic Candida

*Candida albicans*, *Candida glabrata*, *Candida tropicalis*, *Candida parapsilosis*, and *Candida krusei* are the five most prevalent pathogenic *Candida* species [27]. The five *Candida* species were cultured in yeast extract peptone dextrose (YEPD) broth. The antimicrobial test for *Candida* followed the same procedure as for *S. aureus* and *E*. *coli*, using a 100 μM Clotrimazole solution (Thermo Fisher Scientific) as the positive control and YEPD broth as the negative control. Observations were recorded every 24 h over 48 h [28,29].

#### 2.2.3. Symbiosis Properties

The symbiotic properties of *L. plantarum* Probio87 with prevalent vaginal taxa, such as *L. crispatus* and *L. iners* were investigated. *L. iners*, unlike other *Lactobacillus* species, is challenging to cultivate on standard MRS agar due to its complex nutritional requirements [30,31]. Based on pre-experimental findings, BHIs broth was used as an alternative liquid medium for *L. iners*. The analysis method was adapted from the protocol for *S. aureus* and *E. coli*, with modifications including a positive control of 2 mg/L Clindamycin for *L. crispatus* [32] and 1 mg/L Clindamycin for *L. iners* [33]. Observations were recorded at 24-h intervals over 48 h.

### 2.3. Anticancer Assays

#### 2.3.1. Cancer Cell Cultivation

HPV-positive cervical cancer cells (HeLa and CaSki) were cultured in DMEM medium (Nacalai Tesque, Kyoto, Japan), while HPV-negative cervical cancer cells (C-33A) were cultured in DMEM F12 medium (Nacalai Tesque). Both media were supplemented with 10% fetal bovine serum (Tico Europe, Amstelveen, Netherlands) and 1% penicillin-streptomycin (Biowest, Riverside, MO, USA). All cell lines were sub-cultured twice in T75 culture flasks with vented caps and incubated at 37 °C in a 5% CO_2_ atmosphere before being used in experiments.

#### 2.3.2. MTT Array

MTT (3-(4, 5-dimethylthiazol-2-yl)-2, 5 diphenyl tetrazolium bromide) cell viability assay was performed in a 96-well plate format according to the manufacturer’s protocol. HeLa and Caski cells were seeded at 2500 cells per well, while C-33A cells were seeded at 3000 cells per well in 96-well plates overnight. The cells were subsequently treated with a mixture of 30% CFS or vehicle control (VC) and 70% culture medium, and then incubated under standard conditions for 48 h. After the incubation, 10 µL of MTT (5 mg/mL) was added to each well. The plates were then covered with aluminum foil and incubated for an additional 4 h. The medium was then aspirated, and the formazan crystals were solubilized in 100 µL of dimethyl sulfoxide (Merck, Rahway, NJ, USA). The absorbance was measured at 570 nm, and the growth rate was calculated using Equation (3).

(3) $$Growth\;rate\%=\frac{{(OD}_{t}-{OD}_{blank\_t})-({OD}_{0}-{OD}_{blank\_0})}{\left({OD}_{0}-{OD}_{blank\_0}\right)}\times 100\%$$

*OD_t_*: OD measured after 48 h;
*OD*_0_: OD measured before the treatment;
*OD_blank_*: OD of media with no cells at 0 h or after 48 h.

#### 2.3.3. Angiogenesis Array

The angiogenesis assay was performed using the Quantibody^®^ Human Angiogenesis Array 1 (RayBiotech, Peachtree Corners, GA, USA) according to the manufacturer’s instructions. A population of 10,000 HeLa, CaSki, or C-33A cells was plated in each well of a 96-well plate, with 100 μL of complete culture medium. After cell attachment, HeLa and CaSki cell lines were treated for 24 h with a mixture of 30% CFS/VC and 70% culture medium, while the C-33A cell line was treated with a mixture of 10% CFS/VC and 90% culture medium. After treatment, the medium in each well was replaced with 50 μL of serum-free medium, and the cells were incubated for an additional 48 h. The conditioned media were collected and analyzed using a sandwich enzyme-linked immunosorbent assay (ELISA), following the provided protocol, and scanned by the manufacturer. The Gene Array List (GAL) file is available at www.RayBiotech.com/Gal-Files.html (accessed on 10 April 2024) for data extraction. The specialized QAnalyzer tool was also used to analyze the data and generate the results.

#### 2.3.4. Cancer Pathway and Dual Luciferase Test

The impact of Probio87 on key oncogenic signaling pathways was assessed using the Cignal Finder Cancer 10-Pathway Reporter Array (QIAGEN, Hilden, Germany), following the manufacturer’s guidelines. Briefly, 2.5 × 10^4^ cells, prepared in Dulbecco’s Phosphate-Buffered Saline (Sigma-Aldrich), were added to the transfection complex. After 48-h transfection, the transfection medium was substituted with the treatment medium (30% CFS/ VC for HeLa and CaSki cells; 10% CFS/ VC for C-33A cells) and incubated for an extra 24 h. A luciferase assay was performed using the Dual-Glo^®^ Luciferase Assay System, following the manufacturer’s protocol. Briefly, Dual-Glo^®^ Reagent was added to the wells, and the plates were incubated at 25 °C for 30 min in the dark. Firefly luciferase luminescence was then quantified using a CLARIOstar microplate reader (BMG Labtech, Ortenberg, Germany). After a 15-min incubation in the dark with Dual-Glo^®^ Stop & Glo^®^ Reagent, Renilla luciferase luminescence was measured. The relative fold change was calculated using Equation (4). Values greater than 1 indicated upregulation, while values below 1 indicated downregulation of the targeted transcription factors.

(4) $$Relative\;fold\;change\;in\;activity=\frac{Firefly/Renilla\;ratio\;of\;treatment\;group}{Firefly/Renilla\;ratio\;of\;control\;group}$$

#### 2.3.5. Reverse Transcription Quantitative Real-Time PCR

Reverse Transcription Quantitative Polymerase Chain Reaction (RT-qPCR) was used to verify that certain biomarkers link key cancer pathways and inhibit the proliferation of cervical cancer cells. HeLa and CaSki cells were treated for 24 h with a mixture of 30% CFS or VC (MRS broth) in 70% complete cell culture medium, while C-33A cells were treated with 10% CFS or VC. Total RNA was extracted from the treated cells using GENEzol (Geneaid Biotech, New Taipei City, Taiwan), according to the manufacturer’s instructions. The RNA was then reverse transcribed into cDNA using the RevertAid RT Reverse Transcription Kit (Thermo Fisher Scientific). The qPCR was performed using Luna^®^ (2×) SYBR Universal qPCR Master Mix (New England Biolabs, Ipswich, MA, USA) on an Agilent AriaMx Real-time PCR System (Agilent Technologies, Santa Clara, CA, USA). The qPCR protocol included an initial hold at 95 °C for 20 s, followed by 40 cycles of 95 °C for 15 s, 55 °C for 30 s, and 72 °C for 30 s. A melt curve analysis was performed with one cycle at 95 °C for 30 s, 65 °C for 30 s, and a final 95 °C for 30 s. The genes of interest, *ARF* and *P21*, were normalized to the *GAPDH* housekeeping gene, and data were analyzed using the comparative CT (ΔΔCT) method. The primers used for the genes are listed below:

*GAPDH*:

Forward primer: 5′-GTCTCCTCTGACTTCAACAGCG-3′

Reverse primer: 5′-ACCACCCTGTTGCTGTAGCCAA-3′

*ARF:*

Forward primer: 5′-CCCTCGTGCTGATGCTACTG-3′

Reverse primer: 5′-ACCTGGTCTTCTAGGAAGCGG-3′

*P21:*

Forward primer: 5′-GCAGACCAGCATGACAGATTTC-3′

Reverse primer: 5′-CGGATTAGGGCTTCCTCTTG-3′

### 2.4. Statistical Analysis

Data were analyzed using SPSS version 24.0 (SPSS Inc., Chicago, IL, USA). The primary hypothesis of this study involved differential efficacy between the two groups of probiotics and placebo. All tests were two-sided with *p* < 0.05 as considered statistically significant, and data are presented as mean value ± standard deviation unless stated otherwise. Figures were drawn by GraphPad Prism version 8 (GraphPad Software, San Diego, CA, USA). Each experiment was performed in triplicate from three independent batches unless otherwise specified.

## 3. Results

### 3.1. Probiotic Properties

#### 3.1.1. Carbohydrate Utilization

The Analytical Profile Index (API) is a bacterial classification system that employs biochemical tests to facilitate rapid identification [32]. Utilizing the API, the carbohydrate utilization profile of Probio87 was assessed, revealing its ability to metabolize 25 distinct types of carbohydrates (Table 2). Notably, the results remained consistent across both 24 and 48 h of incubation. This carbohydrate utilization pattern corroborated the classification of Probio87 as *Lactiplantibacillus plantarum* [7].

#### 3.1.2. Acid Tolerance

**Figure 1:**
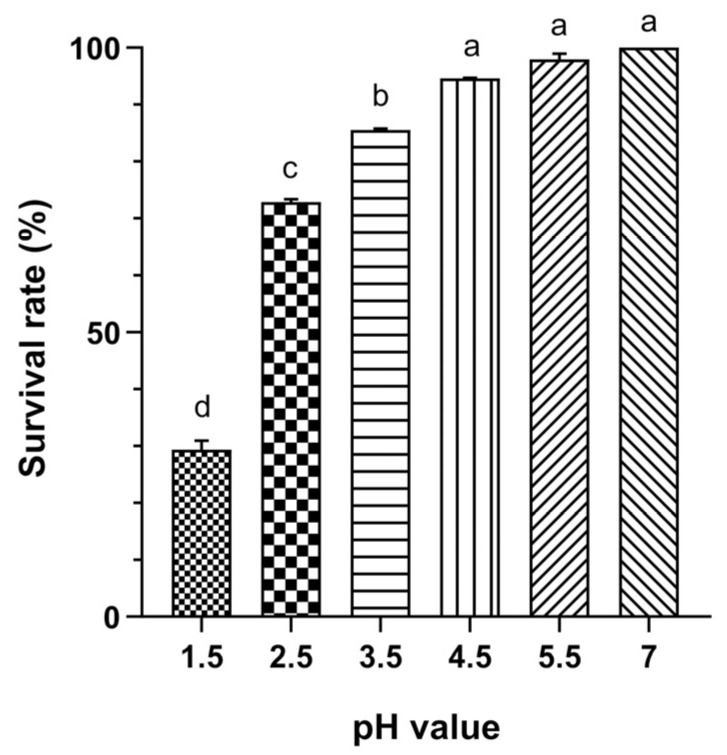
Acid tolerance of *L. plantarum* Probio87. Viability across various pH broths following 4 h of incubation at 37 °C. Data are presented as Mean ± SD. pH 7 was considered the baseline at 100%, with other pH values representing different sample broths. ^a–d^ Significant differences between groups (*p* < 0.05), as determined by one-way ANOVA followed by post hoc multiple comparison tests.

The results indicated that Probio87 exhibits strong acid tolerance, maintaining high survival rates across a range of acidic conditions. It showed excellent viability at pH 4.5 and above, with a survival rate of approximately 86% at pH 3.5 and 73% at pH 2.5. Even at pH 1.5, a highly acidic environment, Probio87 retained about 30% viability (Figure 1). These findings indicate that Probio87 is well-adapted to acidic environments, supporting its potential to survive passage through the stomach.

#### 3.1.3. Bile Tolerance

**Figure 2:**
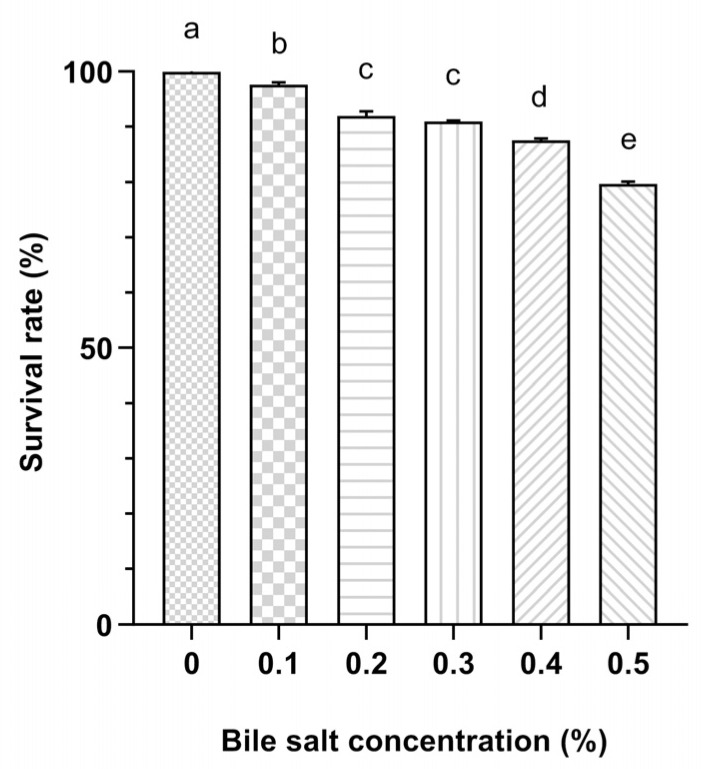
Bile tolerance of *L. plantarum* Probio87. Bile tolerance abilities as demonstrated by the viability of *L. plantarum* Probio87 in broths containing varying concentrations of bile salts after 4 h of incubation at 37 °C. ^a–e^ Significant differences between groups (*p* < 0.05), as determined by one-way ANOVA followed by post hoc multiple comparison tests. Data are presented as Mean ± SD.

In this study, Probio87 demonstrated strong bile salt tolerance, maintaining survival rates above 80% across all tested concentrations from 0% to 0.5%. Notably, it achieved a high survival rate of approximately 91% at 0.3% bile salts, comparable to its performance at 0.2% (Figure 2). These results indicate that Probio87 is well-equipped to withstand bile salt stress, underscoring its potential to survive and function effectively in the gastrointestinal environment.

#### 3.1.4. Antibiotic Resistance

The minimum inhibitory concentrations (MICs) of Probio87 were interpreted according to the cut-off values recommended by the European Food Safety Authority (EFSA) for bacterial antimicrobial susceptibility. Probio87 was susceptible to all seven antibiotics tested for *Lactiplantibacillus plantarum*, with MIC values equal to or below the EFSA-established thresholds (Table 3). These results confirm that Probio87 complies with EFSA safety standards, supporting its suitability as a safe and promising probiotic candidate for human consumption.

#### 3.1.5. Prebiotic Utilization

**Figure 3:**
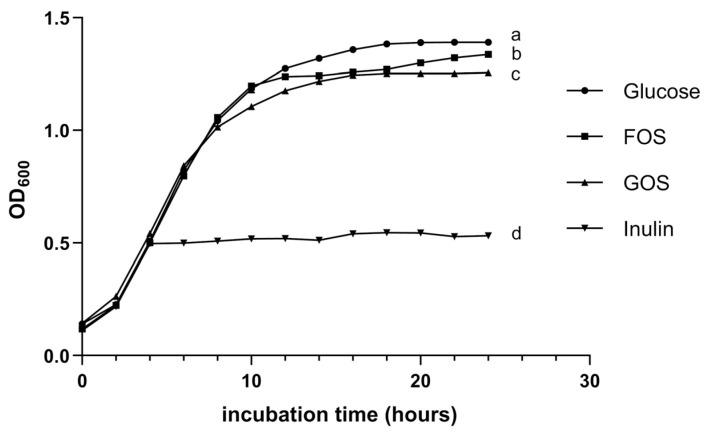
Prebiotic utilization abilities of *L. plantarum* Probio87. The growth was measured by optical density at 600 nm, in the presence of various prebiotics (GOS, FOS, INU, Glu) at 37 °C. Measurements were taken at 2-h intervals over 24 h. Data are presented as Mean ± SD. ^a–d^ Significant differences between groups (*p* < 0.05), as determined by repeated measures ANOVA followed by post hoc multiple comparison tests.

Probio87 showed a strong ability to utilize shorter-chained fructose-based oligosaccharides like fructooligosaccharides (FOS), showing higher growth compared to galactose-based oligosaccharides like galactooligosaccharides (GOS). Although its growth was more limited with longer-chained oligosaccharides, such as inulin (Figure 3), this pattern aligns with the results of the API test. Overall, the capacity of Probio87 to efficiently metabolize key prebiotics is sufficient to support its probiotic properties, contributing to its functionality and survivability in the gut environment.

#### 3.1.6. Adherence to Mucin

In this study, Probio87 exhibited a strong mucin adhesion rate of 75.65% after 3 h of exposure to porcine mucin. Previous research has shown that adhesion capacity among *Lactobacillus* species can vary widely, ranging from as low as 5% in *L. gasseri* S1031 to approximately 70% in *L. fermentum* I5007 [34,35]. The high adhesion observed for Probio87 indicates a pronounced mucin-binding ability within the GIT.

### 3.2. Antimicrobial Properties

#### 3.2.1. Inhibition Against Common Pathogens

**Figure 4:**
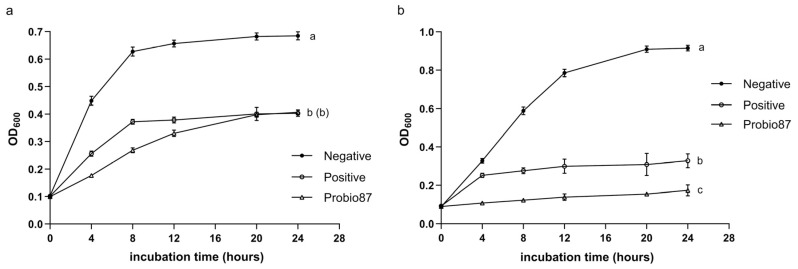
Antimicrobial activity of *L. plantarum* Probio87’s CFS against common pathogens. The CFS of *L. plantarum* Probio87 against (**a**) *S. aureus* and (**b**) *E. coli*. The negative control shows pathogen growth in TSB without treatment. Streptomycin (10 µg/mL) and amoxicillin (1.5 μg/mL) treated pathogens serve as positive controls. Data are presented as Mean ± SD. ^a–c^ Significant differences between groups (*p* < 0.05), as determined by repeated measures ANOVA followed by post hoc multiple comparison tests.

The results showed that the growth of both *Staphylococcus aureus* and *Escherichia coli* was almost completely inhibited after 24 h of treatment with the cell-free supernatant (CFS) of Probio87 (Figure 4). This demonstrates that Probio87 produces bioactive compounds with strong antimicrobial properties capable of effectively suppressing both Gram-positive and Gram-negative pathogens. The potent inhibitory effect, comparable to the positive control, highlights the probiotic’s potential role in pathogen control and maintaining microbial balance in the host environment.

#### 3.2.2. Inhibition Against Pathogenic Candida

**Figure 5:**
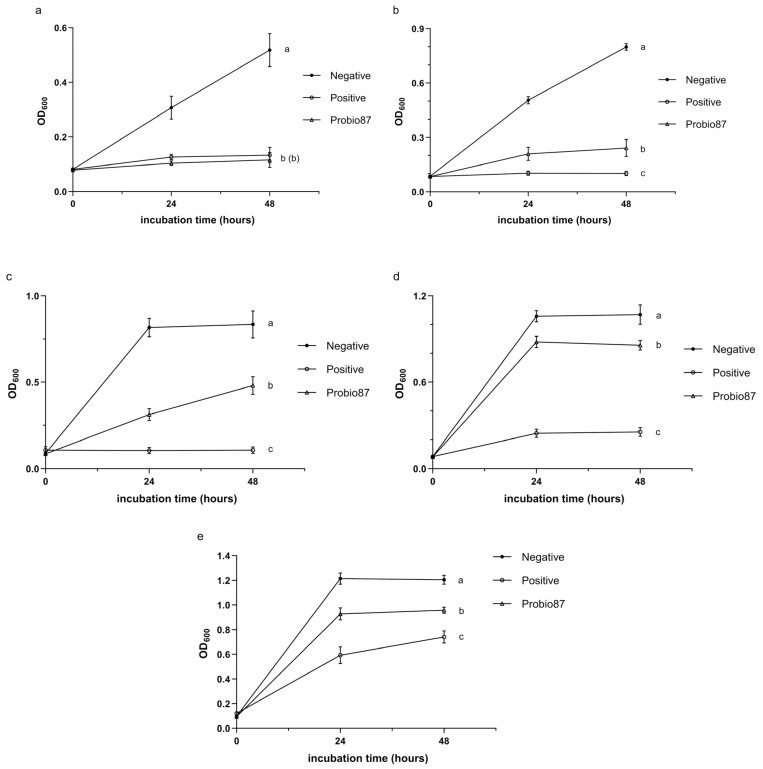
Antimicrobial activity of *L. plantarum* Probio87’s CFS against pathogenic *Candida* species. Antimicrobial activity of *L. plantarum* Probio87’s CFS against (**a**) *C. albicans*, (**b**) *C. parapsilosis*, (**c**) *C. krusei*, (**d**) *C. tropicalis*, and (**e**) *C. glabrata*. The negative control shows pathogen growth in YEPD without treatment. Pathogens treated with Clotrimazole (100 μM) serve as positive controls. Data are presented as Mean ± SD. ^a–c^ Significant differences between groups (*p* < 0.05), as determined by repeated measures ANOVA followed by post hoc multiple comparison tests.

Probio87 demonstrated strong antifungal activity against a range of *Candida* species. It significantly inhibited the growth of *C. parapsilosis* and *C. krusei* (*p* < 0.05; Figure 5b,c), indicating a notable suppressive effect. Against *C. albicans*, one of the most common and virulent fungal pathogens, Probio87 showed inhibitory effects comparable to the positive control (Figure 5a), further highlighting its potent antifungal capacity. Although the inhibition of *C. tropicalis* and *C. glabrata* was less pronounced (Figure 5d,e), Probio87 still exhibited observable antifungal activity.

#### 3.2.3. Symbiosis Properties

**Figure 6:**
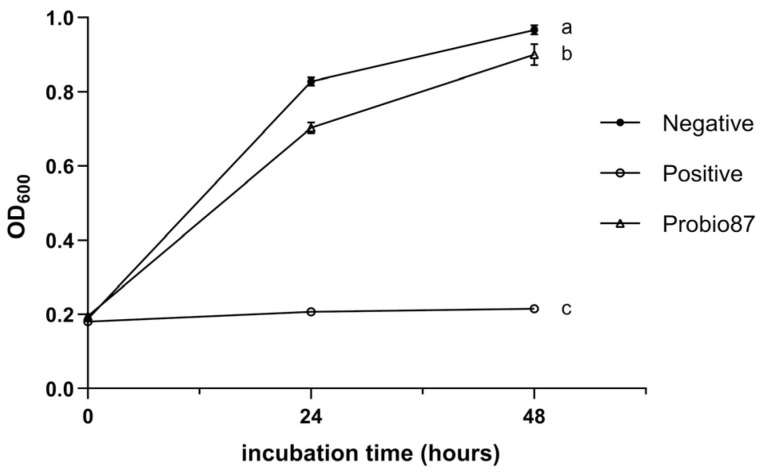
Symbiotic influence of *L. plantarum* Probio87’s CFS on *L. crispatus*. The negative control is the culture medium (MRS broth) without treatment. Clindamycin (2 mg/L) serves as the positive control. Data are presented as Mean ± SD. ^a–c^ Significant differences between groups (*p* < 0.05), as determined by repeated measures ANOVA followed by post hoc multiple comparison tests.

**Figure 7:**
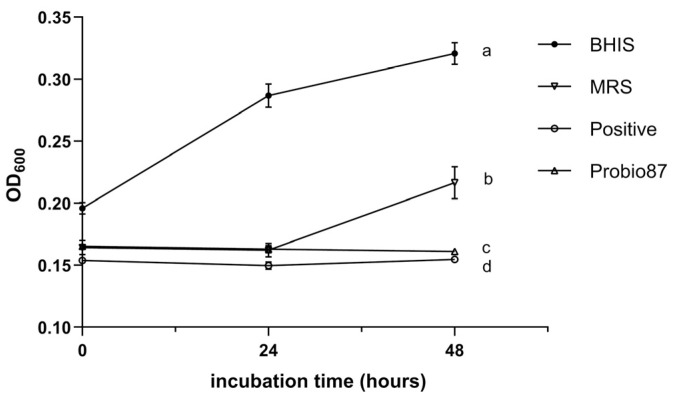
Symbiotic influence of *L. plantarum* Probio87’s CFS on *L. iners*. The negative controls are culture media (BHIs broth and MRS broth). Clindamycin (1 mg/L) serves as the positive control. Data are presented as Mean ± SD. ^a–d^ Significant differences between groups (*p* < 0.05), as determined by repeated measures ANOVA followed by post hoc multiple comparison tests.

Although Probio87 caused a statistically significant inhibition of *L. crispatus* compared to the negative control (MRS broth), the difference observed in the figure was relatively small (Figure 6), suggesting a minimal impact on this beneficial strain. In contrast, Probio87 exhibited a strong inhibitory effect against *L. iners* (Figure 7), comparable to that of the positive control, Clindamycin (1 mg/L).

### 3.3. Anticancer Assays

#### 3.3.1. MTT Assay

**Figure 8:**
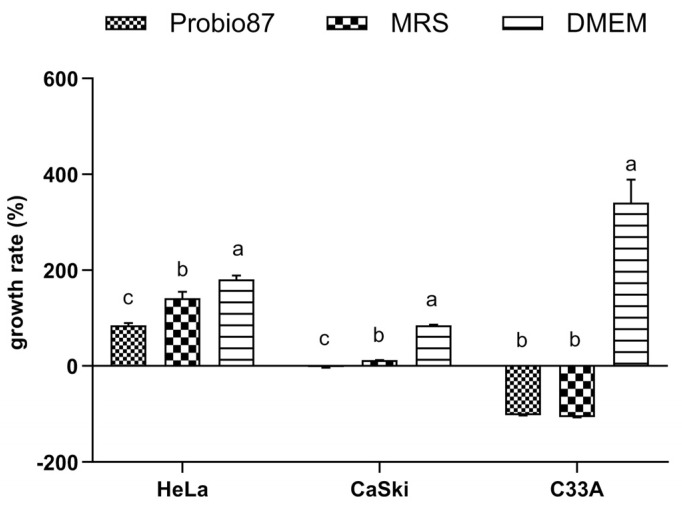
MTT-based analysis of cervical cancer cell growth following treatment with *Lactiplantibacillus plantarum* probio87’s CFS. Cell viability of cervical cancer cell lines—HPV-negative C-33A, HPV-16 positive CaSki, and HPV-18 positive HeLa—after 48-h treatment with the cell-free supernatant (CFS) of *Lactiplantibacillus plantarum* Probio87, Dulbecco’s Modified Eagle Medium (DMEM), or unfermented de Man, Rogosa, and Sharpe (MRS) broth, assessed using the MTT assay. Data are presented as mean ± SD. ^a–c^ Significant differences between groups (*p* < 0.05), as determined by one-way ANOVA followed by post hoc multiple comparison tests.

Treatment with the CFS of Probio87 significantly reduced the growth of HPV-positive cervical cancer cell lines HeLa and CaSki compared to those treated with DMEM or MRS broth (*p* < 0.05; Figure 8). In contrast, the non-HPV C-33A cells exhibited a growth pattern similar to that of the MRS-treated group, with lower proliferation only when compared to the DMEM control (*p* < 0.05). These findings indicate that Probio87 exerts a targeted inhibitory effect, with a more pronounced suppression of HPV-positive cancer cells, highlighting its potential as a probiotic strain with selective anticancer activity.

#### 3.3.2. Angiogenesis Array

**Figure 9:**
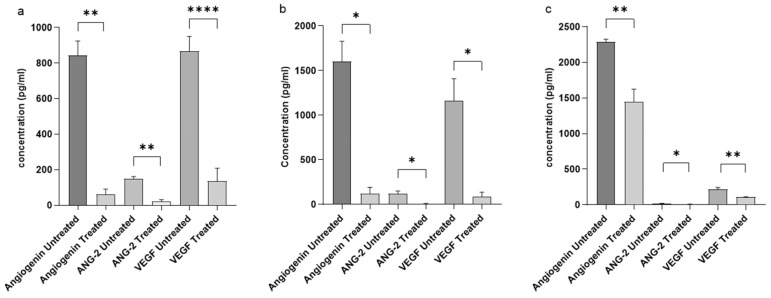
Concentrations of angiogenesis promoters in cervical cancer cell lines following treatment with *Lactiplantibacillus plantarum* Probio87’s CFS. The concentrations of potent angiogenesis promoters in cervical cancer cells (**a**) HeLa, (**b**) CaSki, and (**c**) C-33A cells after a 24-h treatment period with CFS of *L. plantarum* Probio87. Results represented by mean ± SD. * *p* < 0.05, ** *p* < 0.01, **** *p* < 0.0001 via paired *t*-test against the vehicle control.

The CFS of Probio87 significantly downregulated key pro-angiogenic factors—VEGF, ANG-2, and Angiogenin—across all three cervical cancer cell lines compared to the vehicle control (Figure 9). These angiogenic promoters are known to coordinate the stimulation of new blood vessel formation, a process essential for sustaining tumor growth, invasion, and metastasis [36]. The consistent suppression of these markers by Probio87 suggests that it exerts anti-angiogenic effects, potentially impairing the vascular support required for tumor progression. These findings highlight angiogenesis inhibition as a key mechanism by which Probio87 may exert its anticancer activity.

#### 3.3.3. Cancer Pathway and RT-qPCR Test

**Figure 10:**
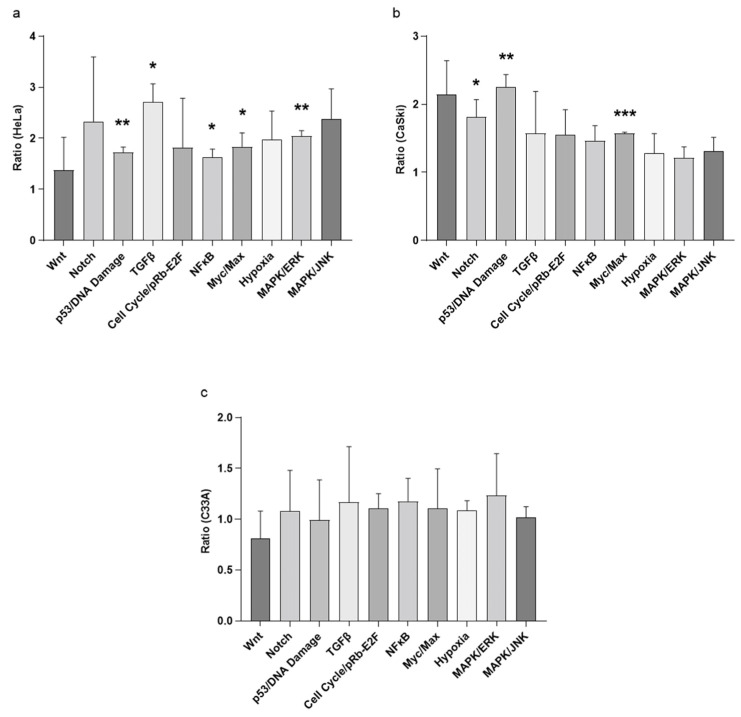
Fold change ratio of transcription factors regulated in three cervical cancer cell lines. Fold change ratio of transcription factors regulated in (**a**) HPV-18 mediated cervical cancer cell HeLa, (**b**) HPV-16 mediated cervical cancer cell CaSki, and (**c**) non-HPV cervical cancer cell C33A upon treatment with CFS of *L. plantarum* Probio87. A value higher than one represented the upregulation of a particular transcription factor, whereas values below one indicated the downregulation of the target. Results represented by mean ± SD. * *p* < 0.05, ** *p* < 0.01, *** *p* < 0.001 via paired *t*-test against the vehicle control.

**Figure 11:**
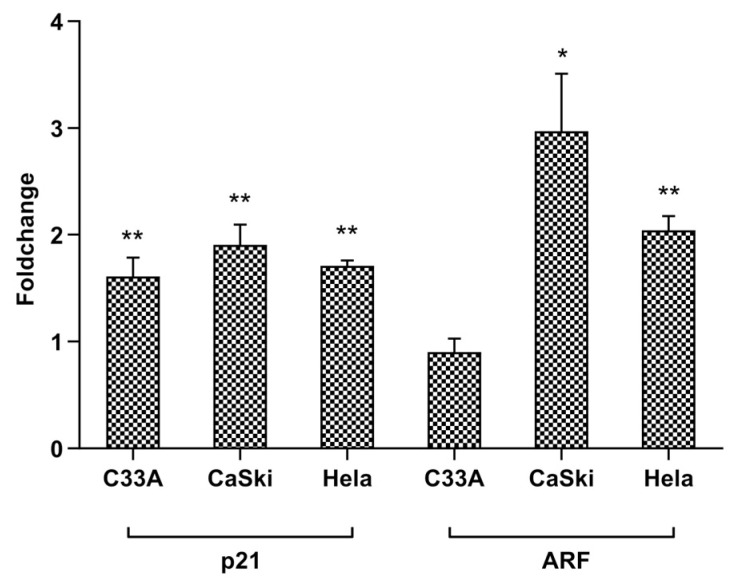
Relative expression levels of *P21* and *ARF* genes in three cervical cancer cell lines. Changes in relative gene expression levels for tumour suppressors *P21* and *ARF* in non-HPV cervical cancer cell C-33A, HPV-16 mediated cervical cancer cell CaSki, and HPV-18 mediated cervical cancer cell HeLa upon treatment with CFS of *L. plantarum* Probio87. Results represented by mean ± SD. * *p* < 0.05, ** *p* < 0.01, using 2^−ΔΔCt^ values via independent *t*-test.

Treatment with the CFS of Probio87 resulted in the activation of multiple antiproliferative and tumor-suppressive signaling pathways in HPV-positive cervical cancer cell lines. In HeLa cells, significant upregulation was observed in the expression of genes associated with the p53, TGF-β, NFκB, Myc/Max, and MAPK/ERK pathways (Figure 10a). Similarly, in CaSki cells, the Notch, p53, and Myc/Max pathways were markedly upregulated following CFS treatment (Figure 10b). In contrast, C-33A cells, which are HPV-negative, exhibited no substantial changes in these signaling cascades (Figure 10c), suggesting a degree of specificity in the response to HPV-transformed cells. Furthermore, RT-qPCR analysis demonstrated a consistent induction of *P21* expression across all three cervical cancer cell lines, indicating a broad mechanism of cell cycle inhibition. Notably, *ARF* was selectively upregulated in the HPV-positive HeLa and CaSki lines, but not in the HPV-negative C-33A cells (Figure 11). These findings support the hypothesis that Probio87 exerts antiproliferative effects through the modulation of oncogenic and tumor-suppressive signaling networks, particularly in HPV-associated cervical carcinoma models.

## 4. Discussion

Evaluating probiotic properties is essential for selecting a viable candidate, as key traits such as acid and bile salt tolerance are critical for survival, colonization, and functional efficacy in the GIT. The GIT presents varying conditions, with the stomach being highly acidic (pH 1.5–3.5) and the colon having a more neutral to slightly acidic environment [37,38]. Effective probiotics must survive the acidic environment of the stomach and the bile salts present in the small intestine. Studies show that many LAB struggle to survive in very acidic environments [39,40]. Probio87 demonstrated a good acid tolerance, maintaining about 30% viability at pH 1.5, 73% at pH 2.5, and 86% at pH 3.5, indicating its potential to survive passage through the stomach. Similarly, bile salt tolerance is essential for probiotic survival in the small intestine, where bile concentration typically reaches 0.3% during digestion [41,42]. Probiotics that withstand bile are more likely to colonize the gut and compete with harmful bacteria [43,44]. Various *L. plantarum* strains exhibit survival rates ranging from 71% to 80% in 0.3% bile salt conditions [45,46], whereas Probio87 demonstrated a higher survival rate of 91% under the same conditions in this study. It showed equal or better performance to strains from previous studies, confirming its strong resistance to acid and bile salts and supporting its potential as an effective probiotic.

Additionally, carbohydrate utilization, prebiotic metabolism, and mucin adhesion play key roles in supporting the growth and functionality of probiotics within the gastrointestinal environment. The classification of Probio87 as *Lactiplantibacillus plantarum* was validated through its carbohydrate utilization profile [8], consistent with its genomic sequencing data. Efficient carbohydrate metabolism, especially of prebiotics, is a critical factor influencing the survival, colonization, and functional integration of probiotics within the host gut microbiota. In this study, Probio87 exhibited a strong ability to utilize fructooligosaccharides (FOS) more efficiently than galactooligosaccharides (GOS), while showing limited growth on inulin. This metabolic preference for shorter-chain fructans is a common trait among clinically established probiotic strains [47,48], supporting its application in synbiotic formulations. The inability to ferment inulin does not undermine its probiotic efficacy, as numerous validated probiotics similarly lack this capability [49,50]. Furthermore, Probio87 exhibited a pronounced ability to adhere to mucin, a critical determinant of gastrointestinal persistence. Adhesion to the mucus layer facilitates prolonged retention, competitive exclusion of pathogens, and sustained immunological engagement. These attributes are particularly relevant for *Lactobacillus* spp., which often exert their beneficial effects through close interaction with the intestinal epithelium [51]. Mucin adhesion assays demonstrated robust binding by Probio87, consistent with FAO/WHO guidelines for probiotic characterization [52]. These results support its potential as an effective mucosal colonizer. Collectively, the efficient fermentation of short-chain fructans, strong mucin-binding, and compatibility with beneficial local bacteria highlight the probiotic potential of Probio87 for synbiotic applications targeting gut microbiota and host–microbe symbiosis.

Probiotics may provide the host with various health benefits, including antimicrobial activity against pathogens. *Staphylococcus aureus* and *Escherichia coli* are prevalent opportunistic pathogens commonly associated with nosocomial infections, foodborne illnesses, and community-acquired infections [53,54]. Their increasing antibiotic resistance, largely driven by overuse and misuse, presents a major challenge to public health [55]. In this study, Probio87 demonstrated significantly greater inhibitory activity against both *S. aureus* and *E. coli* compared to standard antibiotics used as positive controls. Additionally, Probio87 demonstrated selective modulatory effects on *L. crispatus* and *L. iners.* The vaginal microbiota (VM) is commonly categorized into community state types (CSTs), with CST I dominated by *L. crispatus*, a species associated with a reduced risk of urinary tract infections [56], and CST III dominated by *L. iners*, frequently associated with bacterial vaginosis, high-risk HPV infections, and high-grade cervical lesions [57]. *L. crispatus* is widely regarded as a hallmark of vaginal health due to its strong production of lactic acid, hydrogen peroxide, and bacteriocins, which help maintain a low pH and inhibit pathogen colonization [56,58]. In contrast, *L. iners* is often prevalent in transitional or dysbiotic states and lacks several protective functions exhibited by *L. crispatus* [31,59]. Moreover, it expresses potential virulence factors such as aerolysin, a pore-forming toxin similar to vaginolysin produced by *Gardnerella* [60]. The selective inhibition of *L. iners* by Probio87, with minimal impact on *L. crispatus*, suggests a beneficial modulatory effect that may suppress dysbiosis while preserving symbiotic species. This targeted activity highlights the potential of Probio87 as a probiotic agent capable of promoting a more stable and protective VM. In addition to its antibacterial activity, Probio87 exhibited potent antifungal effects against clinically relevant *Candida* species, which are responsible for the majority of fungal infections, particularly in the female urogenital tract. *Candida albicans*, *Candida glabrata*, *Candida tropicalis*, *Candida parapsilosis*, and *Candida krusei* account for over 90% of Human Candidiasis cases, with *C. albicans* being the most predominant [27,61]. The increasing prevalence of *C. glabrata* is particularly concerning due to its reduced sensitivity to azole antifungals [27]. In this study, the CFS of Probio87 effectively inhibited the growth of *Candida* species, demonstrating strong efficacy against *C. albicans* and notable activity against *C. glabrata*. The antifungal activity is likely due to the production of biosurfactants and other bioactive metabolites by Probio87. These compounds can interfere with fungal adhesion and biofilm formation, while also enhancing host epithelial defenses [61,62]. Collectively, these findings demonstrate the broad-spectrum antimicrobial activity of Probio87, which exhibits both antibacterial and antifungal properties and selectively targets potential pathogens. This multifunctional profile positions Probio87 as a promising candidate for therapeutic applications, with the potential to restore microbial homeostasis and manage infections in both the gastrointestinal and urogenital systems.

In the MTT assay, Probio87 CFS significantly inhibited the growth of HeLa and CaSki cells compared to both DMEM and MRS controls (*p* < 0.05), while no such effect was observed in HPV-negative C-33A cells. This suggests that Probio87 CFS selectively inhibits HPV-positive cells but has a neutral effect on HPV-negative C-33A cells. In addition to its cytotoxic effects, Probio87 CFS significantly downregulated key pro-angiogenic factors—VEGF, ANG-2, and Angiogenin—in all three cervical cancer cell lines. VEGF is the central driver of angiogenesis, directly stimulating endothelial cell proliferation, migration, and new blood vessel formation [63]. ANG-2 is another key regulator, involved in vascular remodeling and destabilization, and acts synergistically with VEGF to promote pathological angiogenesis, particularly in tumors [64]. Angiogenin contributes to angiogenesis by promoting ribosomal RNA synthesis and endothelial cell migration [65]. However, its role is generally less prominent than that of VEGF or ANG-2. In this context, Probio87 exhibits a more pronounced antiproliferative effect on HPV-positive HeLa and CaSki cells than on HPV-negative C-33A cells. Collectively, Probio87 suppresses tumor cell proliferation and disrupts angiogenic signaling, potentially limiting the vascular support required for tumor progression. These findings support its potential as a therapeutic probiotic for HPV-related cervical cancers.

The pronounced inhibitory effects of Probio87 on HPV-positive cervical cancer cells suggest underlying molecular mechanisms involving key tumor suppressor pathways, particularly those regulated by p53. The Cancer 10-Pathway Reporter Assay revealed that upregulation of the p53 axis was associated with concomitant increases in the expression of downstream effectors, including p21, Myc/Max, and ARF, indicating coordinated activation of p53-dependent tumor-suppressive pathways. p53, a pivotal transcription factor, induces cell cycle arrest and apoptosis following DNA damage or cellular stress [66]. ARF, stabilized under high c-Myc expression, enhances p53 activity by inhibiting MDM2 and ARF-BP1-mediated degradation [67], while also exerting p53-independent growth suppression via c-Myc interaction [68]. The observed elevation of p53, p21, and ARF (*p* < 0.01) in HeLa and CaSki cells underscores Probio87’s impact on HPV suppression. TGFβ, acting synergistically with p53, further reinforces growth inhibition. It promotes senescence and apoptosis through Smad signaling, telomerase repression, and induction of p21 and p16 [69,70,71]. Cells deficient in p53 show diminished TGFβ responses [72], highlighting the interdependence of these pathways. The concurrent activation of TGFβ and p53 signaling in Probio87-treated HeLa cells supports this coordinated antiproliferative effect. NFκB activation, though context-dependent, can induce p53 transcription and enhance pro-apoptotic gene expression such as PUMA and p21 [73,74,75,76]. In osteosarcoma Saos-2 cells, p53 and NFκB cooperate in apoptosis regulation, with p53 activation enhancing NFκB DNA-binding [77]. Moreover, NFκB can induce apoptosis independently of p53 under stress conditions [78,79]. Co-activation of NFκB and p53 in HeLa cells implies a synergistic apoptotic response to Probio87, potentially triggered by probiotic-induced stress. Similarly, MAPK/ERK signaling may promote apoptosis and senescence through the regulation of p53, p21, and dual-specificity phosphatases (DUSPs) [80,81,82,83,84,85,86,87]. ERK1/2 phosphorylates p53 at Ser15/Thr55, stabilizing it and promoting apoptosis [82]. The observed ERK-p53 co-activation suggests a functional interplay contributing to Probio87’s anticancer effect. Notch signaling, known for its dual role in cancer [88,89], displayed tumor-suppressive behaviour in this study. NOTCH1, upregulated by p53 [90,91,92], induces p21 expression and counters HPV E6 activity via activator protein 1 (AP-1), restoring p53 levels [93]. Enhanced p53 and Notch signaling in CaSki cells suggests that Probio87 modulates this axis to inhibit tumor growth.

**Figure 12:**
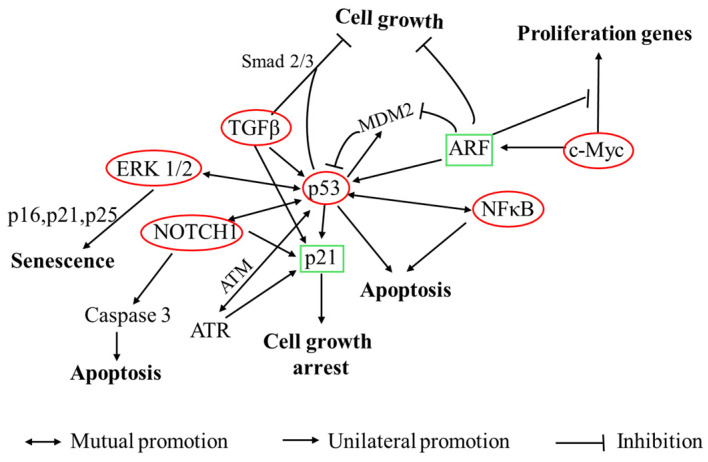
Potential crosstalk among key biomarkers following treatment with probio87’s CFS. The potential interplay of these crucial biomarkers, with p53 assuming a central role. It interacts with the ERK, Notch, and NFκB pathways in a bidirectional manner, where p53 can activate these pathways, and in turn, they can enhance or modulate p53 activity. These pathways can trigger apoptosis or senescence, dependent on or independent of p53. Additionally, c-Myc may induce p53 expression by generating ARF, a robust promoter of apoptosis. TGFβ activation initiates the activation of p53 and p21, which results in cell cycle arrest or apoptosis. Furthermore, TGFβ works synergistically with p53 and Smad 2/3 to inhibit cell growth. The red circles indicate key biomarkers or proteins involved in oncogenic pathways, while the green squares represent important intermediate biomarkers.

Overall, p53 emerges as a central hub integrating Myc/ARF, TGFβ, NFκB, ERK, and Notch pathways (Figure 12). Activation of p53 leads to downstream effects such as increased expression of p21, which mediates cell cycle arrest and apoptosis. Simultaneously, pathways like ARF stabilize p53, TGFβ enhances p53-mediated transcription through Smad signaling, NFκB amplifies p53 transcriptional activity under stress, and ERK1/2 stabilizes p53 through phosphorylation. Moreover, Notch signaling, modulated by p53, promotes p21 expression and counters HPV oncoproteins. These interconnected signaling pathways form a reinforcing regulatory network that amplifies the antiproliferative and pro-apoptotic effects of Probio87, particularly in HPV-positive cervical cancer cells (HeLa, CaSki), compared to HPV-negative cells (C-33A).

## 5. Conclusions

*Lactobacillus* species inhibit pathogens through multiple mechanisms, including competition for adhesion sites and the production of organic acids and bacteriocins. In this study, *Lactiplantibacillus plantarum* Probio87 exhibited key probiotic characteristics, demonstrating strong tolerance to acidic pH and bile salts, mucin adhesion, and efficient utilization of prebiotics such as fructooligosaccharides (FOS) and galactooligosaccharides (GOS). It met EFSA safety criteria by showing no antibiotic resistance, maintaining a carbon metabolism profile consistent with *L. plantarum*, and demonstrating antimicrobial activity against human pathogens while having minimal impact on beneficial symbiotic bacteria. These attributes highlight its strong potential as a probiotic candidate. Additionally, cell culture analysis revealed that the CFS of Probio87 significantly downregulated key angiogenesis promoters, including VEGF, ANG-2, and Angiogenin, across all tested cell lines. Gene expression analysis further revealed that Probio87 treatment upregulated p53-mediated apoptotic pathways, along with the activation of TGFβ, NFκB, Myc/Max, MAPK/ERK, and Notch signaling pathways in HPV-positive cervical cancer cells. Overall, these findings support the dual antimicrobial and anticancer potential of Probio87 and highlight its promise for further clinical investigation.

## Figures and Tables

**Table 1:** Composition of the basal medium supplemented with alternative carbohydrates for prebiotic utilization assays.

Ingredients	Amount (g/L)	Reagent Information
Peptone	10	Bendosen, Selangor Darul Ehsan, Malaysia
Meat extract	8	Himedia, Mumbai, India
Yeast extract	4	
Ammonium citrate	2	
Sodium acetate	3	Oxoid, Basingstoke, Hampshire, UK
Magnesium sulphate	0.1	
Manganese sulphate	0.05	
Dipotassium phosphate	2	
Glu/	20	Bendosen, Selangor Darul Ehsan, Malaysia
FOS/GOS/		NFBC, Yunfu, China
Inulin		Fuji Neihon Seito, Tokyo, Japan

Adjust the pH to 6.2 ± 0.2.

**Table 2:** Carbohydrate utilization profile of *L. plantarum* Probio87, assessed using the API 50 CHL kit after 24 and 48 h of incubation at 37 °C.

Active Ingredients	Reaction	Active Ingredients	Reaction
Control	−	Esculin ferriccitrate	+
Glycerol	−	Salicin	+
Erythritol	−	D-cellobiose	+
D-arabinose	−	D-maltose	+
L-arabinose	+	D-lactose (bovine origin)	+
D-ribose	+	D-melibiose	+
D-xylose	−	D-saccharose	+
L-xylose	−	D-trehalose	+
D-adonitol	−	Inulin	−
Methyl-β-D-xylopyranoside	−	D-melezitose	+
D-galactose	+	D-raffinose	+
D-glucose	+	Amidon (starch)	−
D-fructose	+	Glycogen	−
D-mannose	+	Xylitol	−
L-sorbose	−	Gentiobiose	+
L-rhamnose	−	D-turanose	+
Dulcitol	−	D-lyxose	−
Inositol	−	D-tagatose	−
D-mannitol	+	D-fucose	−
D-sorbitol	+	L-fucose	−
Methyl-α-D-mannopytanoside	+	D-arabitol	−
Methyl-α-D-glucopyranoside	−	L-arabitol	−
*N*-acetylglucosamine	+	Potassium gluconate	+
Amygdalin	+	Potassium 2-ketogluconate	−
Arbutin	+	Potassium 5-ketogluconate	−

+ means the carbohydrate can be used; − means the carbohydrate cannot be used.

**Table 3:** Antibiotic susceptibility profiles of *L. plantarum* Probio87 were determined using the broth microdilution method. The minimum inhibitory concentrations were compared to EFSA susceptibility cut-off values.

Antibiotic	EFSA Cut-Off Values (mg/L)	Probio87 MICs * (mg/L)	Classification
Ampicillin	2	2	Susceptible
Gentamicin	16	1	Susceptible
Kanamycin	64	32	Susceptible
Erythromycin	1	0.06	Susceptible
Clindamycin	2	0.5	Susceptible
Tetracycline	32	32	Susceptible
Chloramphenicol	8	8	Susceptible

* MICs: minimum inhibitory concentrations.

## Data Availability

The raw data supporting the conclusions of this article will be made available by the authors on request.

## Abbreviations

The following abbreviations are used in this manuscript:

ANG-2	angiopoietin-2
API	Analytical Profile Index
ARF	alternate reading frame (tumor suppressor gene)
BHIs	Brain–Heart IUnfusion Supplemented Medium
CFS	cell-free supernatant
CFU	colony-forming unit
CIN	cervical intraepithelial neoplasia
CLSI	Clinical and Laboratory Standards Institute
C-33A	HPV-negative cervical cancer cell line
DMEM	Dulbecco’s Modified Eagle Medium
EFSA	European Food Safety Authority
ELISA	Enzyme-Linked Immunosorbent Assay
FBS	Fetal Bovine Serum
FOS	fructooligosaccharide
GIT	gastrointestinal tract
GAL	gene array list
GOS	galactooligosaccharide
HPV	Human Papillomavirus
HUSM	Hospital Universiti Sains Malaysia
LAB	lactic acid bacteria
LSM	LAB Susceptibility Test Medium
MRS	de Man, Rogosa, and Sharpe Medium
MIC	minimum inhibitory concentration
MTT	3-(4,5-dimethylthiazol-2-yl)-2,5-diphenyl tetrazolium bromide
OD	optical density
PBS	phosphate buffered saline
qPCR	quantitative polymerase chain reaction
RT-qPCR	reverse transcription quantitative polymerase chain reaction
SD	standard deviation
TGF-β	transforming growth factor beta
TSB	Tryptic Soy Broth
VEGF	Vascular Endothelial Growth Factor
VC	vehicle control
VM	vaginal microbiota
YEPD	yeast extract peptone dextrose

## References

[1] Evaluation of Health and Nutritional Properties of Powder Milk and Live Lactic Acid Bacteria. https://www.iqb.es/digestivo/pdfs/probioticos.pdf.

[2] Antimicrobial Resistance. https://www.who.int/news-room/fact-sheets/detail/antimicrobial-resistance.

[3] Mani-López E., Arrioja-Bretón D., López-Malo A. (2022). The Impacts of Antimicrobial and Antifungal Activity of Cell-Free Supernatants from Lactic Acid Bacteria in Vitro and Foods. Compr. Rev. Food Sci. Food Saf..

[4] Mehboudi N., Rahimi H.R., Bakhtiari H.A., Alimardani M., Jalili A. (2023). The Impact of Probiotic Cell-Free Metabolites in MDR Pseudomonas Aeruginosa: Antibacterial Properties and Effect on Antibiotic Resistance Genes Expression. Lett. Appl. Microbiol..

[5] Shi L.H., Balakrishnan K., Thiagarajah K., Mohd Ismail N.I., Yin O.S. (2016). Beneficial Properties of Probiotics. Trop. Life Sci. Res..

[6] Arasu M.V., Al-Dhabi N.A., Ilavenil S., Choi K.C., Srigopalram S. (2016). In Vitro Importance of Probiotic Lactobacillus Plantarum Related to Medical Field. Saudi J. Biol. Sci..

[7] Rudzki L., Ostrowska L., Pawlak D., Małus A., Pawlak K., Waszkiewicz N., Szulc A. (2019). Probiotic Lactobacillus Plantarum 299v Decreases Kynurenine Concentration and Improves Cognitive Functions in Patients with Major Depression: A Double-Blind, Randomized, Placebo Controlled Study. Psychoneuroendocrinology.

[8] Chong H.X., Yusoff N.A.A., Hor Y.-Y., Lew L.-C., Jaafar M.H., Choi S.-B., Yusoff M.S.B., Wahid N., Abdullah M.F.I.L., Zakaria N. (2019). Lactobacillus Plantarum DR7 Alleviates Stress and Anxiety in Adults: A Randomised, Double-Blind, Placebo-Controlled Study. Benef. Microbes.

[9] Cubie H.A. (2013). Diseases Associated with Human Papillomavirus Infection. Virology.

[10] Uehara K., Tanabe Y., Hirota S., Higa S., Toyoda Z., Kurima K., Kina S., Nakasone T., Arasaki A., Kinjo T. (2021). Co-Expression of Low-Risk HPV E6/E7 and EBV LMP-1 Leads to Precancerous Lesions by DNA Damage. BMC Cancer.

[11] Li N., Franceschi S., Howell-Jones R., Snijders P.J., Clifford G.M. (2011). Human Papillomavirus Type Distribution in 30,848 Invasive Cervical Cancers Worldwide: Variation by Geographical Region, Histological Type and Year of Publication. Int. J. Cancer.

[12] Ahmed H.G., Bensumaidea S.H., Alshammari F.D., Alenazi F.S.H., ALmutlaq B.A., Alturkstani M.Z., Aladani I.A. (2017). Prevalence of Human Papillomavirus Subtypes 16 and 18 among Yemeni Patients with Cervical Cancer. Asian Pac. J. Cancer Prev..

[13] Wang Y., Moon A., Huang J., Sun Y., Qiu H.-J. (2022). Antiviral Effects and Underlying Mechanisms of Probiotics as Promising Antivirals. Front. Cell. Infect. Microbiol..

[14] Vanderpool C., Yan F., Polk D.B. (2008). Mechanisms of Probiotic Action: Implications for Therapeutic Applications in Inflammatory Bowel Diseases. Inflamm. Bowel Dis..

[15] Isolauri E., Sütas Y., Kankaanpää P., Arvilommi H., Salminen S. (2001). Probiotics: Effects on Immunity. Am. J. Clin. Nutr..

[16] Moon E.C., Park M.S., Lim T., Kim R.H., Ji G.E., Kim S.Y., Hwang K.T. (2022). Antibacterial Effect of Cell-Free Supernatant Fraction from Lactobacillus Paracasei CH88 against Gardnerella Vaginalis. Sci. Rep..

[17] Li Y., Yu T., Yan H., Li D., Yu T., Yuan T., Rahaman A., Ali S., Abbas F., Dian Z. (2020). Vaginal Microbiota and HPV Infection: Novel Mechanistic Insights and Therapeutic Strategies. Infect. Drug Resist..

[18] Nami Y., Vaseghi Bakhshayesh R., Mohammadzadeh Jalaly H., Lotfi H., Eslami S., Hejazi M.A. (2019). Probiotic Properties of Enterococcus Isolated From Artisanal Dairy Products. Front. Microbiol..

[19] Lee J.-E., Lee N.-K., Paik H.-D. (2020). Antimicrobial and Anti-Biofilm Effects of Probiotic Lactobacillus Plantarum KU200656 Isolated from Kimchi. Food Sci. Biotechnol..

[20] Balouiri M., Sadiki M., Ibnsouda S.K. (2016). Methods for in Vitro Evaluating Antimicrobial Activity: A Review. J. Pharm. Anal..

[21] Klare I., Konstabel C., Werner G., Huys G., Vankerckhoven V., Kahlmeter G., Hildebrandt B., Müller-Bertling S., Witte W., Goossens H. (2007). Antimicrobial Susceptibilities of Lactobacillus, Pediococcus and Lactococcus Human Isolates and Cultures Intended for Probiotic or Nutritional Use. J. Antimicrob. Chemother..

[22] Tham C.S.-C., Peh K.-K., Bhat R., Liong M.-T. (2012). Probiotic Properties of Bifidobacteria and Lactobacilli Isolated from Local Dairy Products. Ann. Microbiol..

[23] Carasi P., Ambrosis N.M., De Antoni G.L., Bressollier P., Urdaci M.C., de los Angeles Serradell M. (2014). Adhesion Properties of Potentially Probiotic *Lactobacillus kefiri* to Gastrointestinal Mucus. J. Dairy Res..

[24] Hütt P., Lapp E., Štšepetova J., Smidt I., Taelma H., Borovkova N., Oopkaup H., Ahelik A., Rööp T., Hoidmets D. (2016). Characterisation of Probiotic Properties in Human Vaginal Lactobacilli Strains. Microb. Ecol. Health Dis..

[25] Pabari K., Pithva S., Kothari C., Purama R.K., Kondepudi K.K., Vyas B.R.M., Kothari R., Ambalam P. (2020). Evaluation of Probiotic Properties and Prebiotic Utilization Potential of Weissella Paramesenteroides Isolated From Fruits. Probiotics Antimicrob. Proteins.

[26] Hor Y.Y., Liong M.T. (2014). Use of Extracellular Extracts of Lactic Acid Bacteria and Bifidobacteria for the Inhibition of Dermatological Pathogen Staphylococcus Aureus. Dermatol. Sin..

[27] Turner S.A., Butler G. (2014). The Candida Pathogenic Species Complex. Cold Spring Harb. Perspect. Med..

[28] Yang X., Chang T., Yuan Q., Wei W., Wang P., Song X., Yuan H. (2022). Changes in the Composition of Gut and Vaginal Microbiota in Patients with Postmenopausal Osteoporosis. Front. Immunol..

[29] Nisaa A.A., Oon C.-E., Sreenivasan S., Balakrishnan V., Tan J.J., Teh C.S.-J., Sany S., Todorov S.D., Liu G., Park Y.-H. (2023). Breast Milk from Healthy Women Has Higher Anti-Candida Properties than Women with Vaginal Infections during Pregnancy. Food Sci. Biotechnol..

[30] Yoshimura K., Ogawa M., Saito M. (2020). In Vitro Characteristics of Intravaginal Lactobacilli; Why Is *L. iners* Detected in Abnormal Vaginal Microbial Flora?. Arch. Gynecol. Obstet..

[31] Zheng N., Guo R., Wang J., Zhou W., Ling Z. (2021). Contribution of Lactobacillus Iners to Vaginal Health and Diseases: A Systematic Review. Front. Cell. Infect. Microbiol..

[32] Happel A.-U., Kullin B., Gamieldien H., Wentzel N., Zauchenberger C.Z., Jaspan H.B., Dabee S., Barnabas S.L., Jaumdally S.Z., Dietrich J. (2020). Exploring Potential of Vaginal Lactobacillus Isolates from South African Women for Enhancing Treatment for Bacterial Vaginosis. PLoS Pathog..

[33] Petrina M.A.B., Cosentino L.A., Rabe L.K., Hillier S.L. (2017). Susceptibility of Bacterial Vaginosis (BV)-Associated Bacteria to Secnidazole Compared to Metronidazole, Tinidazole and Clindamycin. Anaerobe.

[34] Li X.J., Yue L.Y., Guan X.F., Qiao S.Y. (2008). The Adhesion of Putative Probiotic Lactobacilli to Cultured Epithelial Cells and Porcine Intestinal Mucus. J. Appl. Microbiol..

[35] Martín V., Cárdenas N., Ocaña S., Marín M., Arroyo R., Beltrán D., Badiola C., Fernández L., Rodríguez J.M. (2019). Rectal and Vaginal Eradication of Streptococcus Agalactiae (GBS) in Pregnant Women by Using Lactobacillus Salivarius CECT 9145, A Target-Specific Probiotic Strain. Nutrients.

[36] Eelen G., Treps L., Li X., Carmeliet P. (2020). Basic and Therapeutic Aspects of Angiogenesis Updated. Circ. Res..

[37] Xie G., Wang X., Jiang R., Zhao A., Yan J., Zheng X., Huang F., Liu X., Panee J., Rajani C. (2018). Dysregulated Bile Acid Signaling Contributes to the Neurological Impairment in Murine Models of Acute and Chronic Liver Failure. EBioMedicine.

[38] Yuan W., Seng Z.J., Kohli G.S., Yang L., Yuk H.-G. (2018). Stress Resistance Development and Genome-Wide Transcriptional Response of Escherichia Coli O157:H7 Adapted to Sublethal Thymol, Carvacrol, and Trans-Cinnamaldehyde. Appl. Environ. Microbiol..

[39] Gómez N.C., Ramiro J.M.P., Quecan B.X.V., de Melo Franco B.D.G. (2016). Use of Potential Probiotic Lactic Acid Bacteria (LAB) Biofilms for the Control of Listeria Monocytogenes, Salmonella Typhimurium, and Escherichia Coli O157:H7 Biofilms Formation. Front. Microbiol..

[40] Cheon M.-J., Lim S.-M., Lee N.-K., Paik H.-D. (2020). Probiotic Properties and Neuroprotective Effects of Lactobacillus Buchneri KU200793 Isolated from Korean Fermented Foods. Int. J. Mol. Sci..

[41] Gilliland S.E., Staley T.E., Bush L.J. (1984). Importance of Bile Tolerance of Lactobacillus Acidophilus Used as a Dietary Adjunct. J. Dairy Sci..

[42] Zaghloul E.H., Ibrahim M.I.A. (2022). Production and Characterization of Exopolysaccharide From Newly Isolated Marine Probiotic Lactiplantibacillus Plantarum EI6 With in Vitro Wound Healing Activity. Front. Microbiol..

[43] Ruiz L., Margolles A., Sánchez B. (2013). Bile Resistance Mechanisms in Lactobacillus and Bifidobacterium. Front. Microbiol..

[44] Xia Y., Wang M., Gao F., Lu M., Chen G. (2020). Effects of Dietary Probiotic Supplementation on the Growth, Gut Health and Disease Resistance of Juvenile Nile Tilapia (Oreochromis Niloticus). Anim. Nutr..

[45] Zhao X., Zhao C., Yang L., Jiang L., Zhang J., Yu X., Chen G., Zhu H., Tang W., Li Y. (2022). Spatial and Temporal Persistence of Fluorescent Lactiplantibacillus Plantarum RS-09 in Intestinal Tract. Front. Microbiol..

[46] Bu Y., Liu Y., Liu Y., Wang S., Liu Q., Hao H., Yi H. (2022). Screening and Probiotic Potential Evaluation of Bacteriocin-Producing Lactiplantibacillus Plantarum In Vitro. Foods.

[47] Ke A., Parreira V.R., Farber J.M., Goodridge L. (2022). Inhibition of Cronobacter Sakazakii in an Infant Simulator of the Human Intestinal Microbial Ecosystem Using a Potential Synbiotic. Front. Microbiol..

[48] Suissa R., Oved R., Maan H., Hadad U., Gilhar O., Meijler M.M., Koren O., Kolodkin-Gal I. (2022). Context-Dependent Differences in the Functional Responses of Lactobacillaceae Strains to Fermentable Sugars. Front. Microbiol..

[49] Succi M., Tremonte P., Pannella G., Tipaldi L., Cozzolino A., Romaniello R., Sorrentino E., Coppola R. (2017). Pre-Cultivation with Selected Prebiotics Enhances the Survival and the Stress Response of Lactobacillus Rhamnosus Strains in Simulated Gastrointestinal Transit. Front. Microbiol..

[50] Dong Y., Han M., Fei T., Liu H., Gai Z. (2024). Utilization of Diverse Oligosaccharides for Growth by Bifidobacterium and Lactobacillus Species and Their in Vitro Co-Cultivation Characteristics. Int. Microbiol..

[51] Nishiyama K., Sugiyama M., Mukai T. (2016). Adhesion Properties of Lactic Acid Bacteria on Intestinal Mucin. Microorganisms.

[52] Binda S., Hill C., Johansen E., Obis D., Pot B., Sanders M.E., Tremblay A., Ouwehand A.C. (2020). Criteria to Qualify Microorganisms as “Probiotic” in Foods and Dietary Supplements. Front. Microbiol..

[53] Igbinosa I.H., Aighewi I.T. (2022). Detection of Potential Pathogenic Bacteria in Selected Public Swimming Pools: Public Health Implications. Niger. J. Life Sci..

[54] Zhu A., Ali S., Wang Z., Xu Y., Lin R., Jiao T., Ouyang Q., Chen Q. (2023). ZnO@Ag-Functionalized Paper-Based Microarray Chip for SERS Detection of Bacteria and Antibacterial and Photocatalytic Inactivation. Anal. Chem..

[55] Haindongo E.H., Ndakolo D., Hedimbi M., Vainio O., Hakanen A., Vuopio J. (2023). Antimicrobial Resistance Prevalence of *Escherichia Coli* and *Staphylococcus Aureus* amongst Bacteremic Patients in Africa: A Systematic Review. J. Glob. Antimicrob. Resist..

[56] Komesu Y.M., Dinwiddie D.L., Richter H.E., Lukacz E.S., Sung V.W., Siddiqui N.Y., Zyczynski H.M., Ridgeway B., Rogers R.G., Arya L.A. (2020). Defining the Relationship Between Vaginal and Urinary Microbiomes. Am. J. Obstet. Gynecol..

[57] Srinivasan S., Hoffman N.G., Morgan M.T., Matsen F.A., Fiedler T.L., Hall R.W., Ross F.J., McCoy C.O., Bumgarner R., Marrazzo J.M. (2012). Bacterial Communities in Women with Bacterial Vaginosis: High Resolution Phylogenetic Analyses Reveal Relationships of Microbiota to Clinical Criteria. PLoS ONE.

[58] Liu Y., Zhao X., Wu F., Chen J., Luo J., Wu C., Chen T. (2024). Effectiveness of Vaginal Probiotics Lactobacillus Crispatus Chen-01 in Women with High-Risk HPV Infection: A Prospective Controlled Pilot Study. Aging.

[59] The Role of *L. iners* in Vaginal Health: Insights and Controversies. https://ipa-biotics.org/the-role-of-l-iners-in-vaginal-health-insights-and-controversies/.

[60] Bonnardel F., Haslam S.M., Dell A., Feizi T., Liu Y., Tajadura-Ortega V., Akune Y., Sykes L., Bennett P.R., MacIntyre D.A. (2021). Proteome-Wide Prediction of Bacterial Carbohydrate-Binding Proteins as a Tool for Understanding Commensal and Pathogen Colonisation of the Vaginal Microbiome. NPJ Biofilms Microbiomes.

[61] Divyashree S., Shruthi B., Vanitha P.R., Sreenivasa M.Y. (2023). Probiotics and Their Postbiotics for the Control of Opportunistic Fungal Pathogens: A Review. Biotechnol. Rep..

[62] Ribeiro F.C., Rossoni R.D., de Barros P.P., Santos J.D., Fugisaki L.R.O., Leão M.P.V., Junqueira J.C. (2020). Action Mechanisms of Probiotics on Candida Spp. and Candidiasis Prevention: An Update. J. Appl. Microbiol..

[63] Lee C., Kim M.-J., Kumar A., Lee H.-W., Yang Y., Kim Y. (2025). Vascular Endothelial Growth Factor Signaling in Health and Disease: From Molecular Mechanisms to Therapeutic Perspectives. Signal Transduct. Target. Ther..

[64] Hashizume H., Falcón B.L., Kuroda T., Baluk P., Coxon A., Yu D., Bready J.V., Oliner J.D., McDonald D.M. (2010). Complementary Actions of Inhibitors of Angiopoietin-2 and VEGF on Tumor Angiogenesis and Growth. Cancer Res..

[65] Li S., Hu G. (2010). Angiogenin-Mediated rRNA Transcription in Cancer and Neurodegeneration. Int. J. Biochem. Mol. Biol..

[66] Ozaki T., Nakagawara A. (2011). Role of P53 in Cell Death and Human Cancers. Cancers.

[67] Chen D., Kon N., Zhong J., Zhang P., Yu L., Gu W. (2013). Differential Effects on ARF Stability by Normal versus Oncogenic Levels of C-Myc Expression. Mol. Cell.

[68] Datta A., Nag A., Pan W., Hay N., Gartel A.L., Colamonici O., Mori Y., Raychaudhuri P. (2004). Myc-ARF (Alternate Reading Frame) Interaction Inhibits the Functions of Myc. J. Biol. Chem..

[69] Elston R., Inman G.J. (2012). Crosstalk between P53 and TGF-β Signalling. J. Signal Transduct..

[70] Adorno M., Cordenonsi M., Montagner M., Dupont S., Wong C., Hann B., Solari A., Bobisse S., Rondina M.B., Guzzardo V. (2009). A Mutant-P53/Smad Complex Opposes P63 to Empower TGFβ-Induced Metastasis. Cell.

[71] Mikuła-Pietrasik J., Rutecki S., Ksiazek K. (2022). The Functional Multipotency of Transforming Growth Factor β Signaling at the Intersection of Senescence and Cancer. Cell. Mol. Life Sci..

[72] Cordenonsi M., Dupont S., Maretto S., Insinga A., Imbriano C., Piccolo S. (2003). Links between Tumor Suppressors: P53 Is Required for TGF-β Gene Responses by Cooperating with Smads. Cell.

[73] Huang R.-F.S., Wei Y.-J., Inbaraj B.S., Chen B.-H. (2015). Inhibition of Colon Cancer Cell Growth by Nanoemulsion Carrying Gold Nanoparticles and Lycopene. Int. J. Nanomed..

[74] Dey A., Tergaonkar V., Lane D.P. (2008). Double-Edged Swords as Cancer Therapeutics: Simultaneously Targeting P53 and NF-kappaB Pathways. Nat. Rev. Drug Discov..

[75] Wu H., Lozano G. (1994). NF-Kappa B Activation of P53. A Potential Mechanism for Suppressing Cell Growth in Response to Stress. J. Biol. Chem..

[76] Fujioka S., Schmidt C., Sclabas G.M., Li Z., Pelicano H., Peng B., Yao A., Niu J., Zhang W., Evans D.B. (2004). Stabilization of P53 Is a Novel Mechanism for Proapoptotic Function of NF-kappaB. J. Biol. Chem..

[77] Carrà G., Lingua M., Maffeo B., Taulli R., Morotti A. (2020). P53 vs NF-κB: The Role of Nuclear Factor-Kappa B in the Regulation of P53 Activity and Vice Versa. Cell Mol. Life Sci..

[78] Barkett M., Gilmore T.D. (1999). Control of Apoptosis by Rel/NF-kappaB Transcription Factors. Oncogene.

[79] Yin L., Yu X. (2018). Arsenic-Induced Apoptosis in the P53-Proficient and P53-Deficient Cells through Differential Modulation of NFkB Pathway. Food Chem. Toxicol..

[80] Guo Y.-J., Pan W.-W., Liu S.-B., Shen Z.-F., Xu Y., Hu L.-L. (2020). ERK/MAPK Signalling Pathway and Tumorigenesis. Exp. Ther. Med..

[81] Lu Y., Liu B., Liu Y., Yu X., Cheng G. (2020). Dual Effects of Active ERK in Cancer: A Potential Target for Enhancing Radiosensitivity (Review). Oncol. Lett..

[82] Sugiura R., Satoh R., Takasaki T. (2021). ERK: A Double-Edged Sword in Cancer. ERK-Dependent Apoptosis as a Potential Therapeutic Strategy for Cancer. Cells.

[83] Zou J., Lei T., Guo P., Yu J., Xu Q., Luo Y., Ke R., Huang D. (2019). Mechanisms Shaping the Role of ERK1/2 in Cellular Senescence (Review). Mol. Med. Rep..

[84] Ussar S., Voss T. (2004). MEK1 and MEK2, Different Regulators of the G1/S Transition. J. Biol. Chem..

[85] Lin A.W., Barradas M., Stone J.C., van Aelst L., Serrano M., Lowe S.W. (1998). Premature Senescence Involving P53 and P16 Is Activated in Response to Constitutive MEK/MAPK Mitogenic Signaling. Genes Dev..

[86] Cammarano M.S., Nekrasova T., Noel B., Minden A. (2005). Pak4 Induces Premature Senescence via a Pathway Requiring p16INK4/p19ARF and Mitogen-Activated Protein Kinase Signaling. Mol. Cell. Biol..

[87] Zhuang D., Mannava S., Grachtchouk V., Tang W.-H., Patil S., Wawrzyniak J.A., Berman A.E., Giordano T.J., Prochownik E.V., Soengas M.S. (2008). C-MYC Overexpression Is Required for Continuous Suppression of Oncogene-Induced Senescence in Melanoma Cells. Oncogene.

[88] Ntziachristos P., Lim J.S., Sage J., Aifantis I. (2014). From Fly Wings to Targeted Cancer Therapies: A Centennial for Notch Signaling. Cancer Cell.

[89] Shi Q., Xue C., Zeng Y., Yuan X., Chu Q., Jiang S., Wang J., Zhang Y., Zhu D., Li L. (2024). Notch Signaling Pathway in Cancer: From Mechanistic Insights to Targeted Therapies. Signal Transduct. Target. Ther..

[90] Wei C.-L., Wu Q., Vega V.B., Chiu K.P., Ng P., Zhang T., Shahab A., Yong H.C., Fu Y., Weng Z. (2006). A Global Map of P53 Transcription-Factor Binding Sites in the Human Genome. Cell.

[91] Lefort K., Mandinova A., Ostano P., Kolev V., Calpini V., Kolfschoten I., Devgan V., Lieb J., Raffoul W., Hohl D. (2007). Notch1 Is a P53 Target Gene Involved in Human Keratinocyte Tumor Suppression through Negative Regulation of ROCK1/2 and MRCKalpha Kinases. Genes Dev..

[92] Yugawa T., Handa K., Narisawa-Saito M., Ohno S., Fujita M., Kiyono T. (2007). Regulation of Notch1 Gene Expression by P53 in Epithelial Cells. Mol. Cell Biol..

[93] Dotto G.P. (2009). Crosstalk of Notch with P53 and P63 in Cancer Growth Control. Nat. Rev. Cancer.

